# Transcriptional and in silico analyses of MIF cytokine and TLR signalling interplay in the LPS inflammatory response of *Ciona robusta*

**DOI:** 10.1038/s41598-020-68339-x

**Published:** 2020-07-09

**Authors:** Vincenzo Arizza, Angela Bonura, Laura La Paglia, Alfonso Urso, Annalisa Pinsino, Aiti Vizzini

**Affiliations:** 1 Dipartimento di Scienze e Tecnologie Biologiche, Chimiche e Farmaceutiche, Università di Palermo, Via Archirafi 18, Palermo, Italy; 2 Istituto per la Ricerca e l’Innovazione Biomedica–Consiglio Nazionale delle Ricerche, Via Ugo la Malfa 153, Palermo, Italy; 3 Istituto di Calcolo e Reti ad Alte Prestazioni–Consiglio Nazionale delle Ricerche, Via Ugo la Malfa 153, Palermo, Italy

**Keywords:** Inflammation, Computational biology and bioinformatics, Cellular signalling networks, Computational models, Gene ontology, High-throughput screening, Phylogeny

## Abstract

The close phylogenetic relationship between *Ciona robusta* and vertebrates makes it a powerful model for studying innate immunity and the evolution of immune genes. To elucidate the nature and dynamics of the immune response, the molecular mechanisms by which bacterial infection is detected and translated into inflammation and how potential pattern recognition receptors (PRRs) are involved in pathogen recognition in tunicate *C. robusta* (formerly known as *Ciona intestinalis*), we applied an approach combining bacterial infections, next-generation sequencing, qRT-PCR, bioinformatics and in silico analyses (criteria of a p-value < 0.05 and FDR < 0.05). A STRING analysis indicated a functional link between components of the Tlr/MyD88-dependent signalling pathway (Tlr2, MyD88, and Irak4) and components of the Nf-κB signalling pathway (Nf-κB, IκBα, and Ikkα) (p-value < 0.05, FDR < 0.05). A qRT-PCR analysis of immune genes selected from transcriptome data revealed *Mif* as more frequently expressed in the inflammatory response than inflammation mediator or effector molecules (e.g., Il-17s, Tnf-α, Tgf-β, Mmp9, Tlrs, MyD88, Irak4, Nf-κB, and galectins), suggesting close interplay between Mif cytokines and Nf-κB signalling pathway components in the biphasic activation of the inflammatory response. An in silico analyses of the 3′-UTR of *Tlr2, MyD88, IκBα, Ikk*, and *Nf-κB* transcripts showed the presence of GAIT elements, which are known to play key roles in the regulation of immune gene-specific translation in humans. These findings provide a new level of understanding of the mechanisms involved in the regulation of the *C. robusta* inflammatory response induced by LPS and suggest that in *C. robusta,* as in humans, a complex transcriptional and post-transcriptional control mechanism is involved in the regulation of several inflammatory genes.

## Introduction

The innate immune system is an integrated system of biological processes and structures in vertebrates, invertebrates and plants that recognise and fight pathogens^1,2^. This defence mechanism relies on several classes of immune receptors, collectively named pattern recognition receptors (PRRs), which sense pathogen-associated molecular patterns (PAMPs) and induce sophisticated signalling and behaviours, based on dynamic feedback-regulated interactions among a number of components (genes, transcripts, metabolites and proteins). PAMPs include lipopolysaccharides (LPSs), bacterial lipoproteins and lipopeptides, peptidoglycans, mannose-rich glycans, flagellin, bacterial and viral nucleic acids, double stranded (ds) and single stranded (ss) RNA^3^. Among the PRRs, Toll-like receptors (TLRs) the most ancient family, recognize the most extensive spectrum of pathogens^4^.

In recent decades, *Ciona robusta*, the closest living relative of vertebrates, has become a model in various fields of biology, serving as a particularly powerful model for studying innate immunity^5–8^. These chordate invertebrates are equipped with an innate immune system that trigger humoral and cellular responses^9^. It has been widely proven that in *C. robusta*^10–13^, LPS induces an inflammatory response in the pharynx (a haematopoietic organ)^14–18^. *C. robusta* has three *TLR* genes, and only two, called *Tlr1 and Tlr2*, have been cloned^19^. They present “hybrid” functionality, reflecting mammalian TLRs, and are canonically divided into two subfamilies that primarily recognise related PAMPs: (i) TLR1, TLR2, TLR4 and TLR6 recognise lipids, whereas (ii) TLR3, TLR7, TLR8 and TLR9 recognise nucleic acids^20,21^. TLR signalling leads to cell signalling cascades through the myeloid differentiation primary response 88 protein (MyD88), the first molecule activated in the TLR signalling pathway^22,23^ and a central node in inflammatory pathways^24^. MyD88 binds IL-1R-associated kinase family (IRAK) members through one of its well-conserved Toll-interleukin-1-receptor domains, thus leading to the activation of nuclear factor-kappa B (NF-κB)^25^, which, in turn, leads to the expression of proinflammatory cytokines, such as tumour necrosis factor-α (TNF-α) and interferon (IFN)^21^. The NF-κB transcription factor family, which is highly conserved from *Drosophila* to humans, includes five members, RelA/p65, c-Rel, RelB, NF-κB1 (p50), and NF-κB2 (p52), which form homo- and heterodimers. A highly conserved DNA-binding/dimerization domain called the Rel homology domain (RHD) is involved in the formation of Rel/NF-κB dimer proteins^25,26^. Normally, NF-κB proteins are stored and sequestered in the cytoplasm by κB family members, characterised by Ankyrin (ANK) repeats and a solenoid fold, which are usually implicated in specific protein–protein interactions^25,26^. Two major signalling pathways are involved in the activation of NF-κB: the canonical and non-canonical (or alternative) pathways are both involved in regulating immune and inflammatory responses^27–29^. In the canonical pathway in the absence of stimuli, NF-κB transcription factors are bound to IκBs, sequestered in the cytoplasm, with consistently inactive transcription. An appropriate stimulus activates a protein kinase complex (IKK), consisting of IKKα, IKKβ and IKKγ in humans, and in turn phosphorylates IκB, leading to complex degradation and allowing the translocation of NF-κB complexes (predominantly the p50/RelA dimer) from the cytoplasm to the nucleus^30,31^. Non-canonical NF-κB activation does not require IκBα degradation but depends on the processing of the NF-κB2 precursor protein p100, which has yet to be identified in invertebrate organisms^27^. In *C. robusta*, one gene encodes the respective *Nf-κB*, *IκB*, and *Ikk* proteins, and two different isoforms of *Rel* have been identified^32,33^.

In recent years, the number of functions attributed to the human macrophage migration inhibitory factor (MIF) cytokine has increased significantly, positioning MIF as among the most frequently expressed proteins in the inflammatory process^34,35^. MIF is produced and stored intracellularly in different cells of the immune system, including monocytes, macrophages and T cells, in the pituitary gland^36^. MIF has received substantial attention as a mediator of innate and adaptive immune responses^37^, and it has been implicated in many infectious, inflammatory and immune diseases, including rheumatoid arthritis, atherosclerosis and tumorigenesis^38–41^. MIF effects are mediated via an autocrine/paracrine signalling pathways, leading to the activation of ERK1/ERK2 MAP kinases, triggering pro-inflammatory gene (e.g., *TNFα, IL-1*, *IL-6*, *IL-8*, and *IL-12*) and matrix metalloprotease gene expression and upregulating *TLR4* expression^35,41–44^. Recently, two macrophage migration inhibitory factor (*Mif*) genes in *C. robusta* have been cloned: *Mif1* and *Mif2*^45^.

Although genes known to be involved in the immune response, such as *TLR, NF-κB* and *MIF,* are expressed in *C. robusta*, the wide-ranging nature and temporal dynamics of immune signalling in *C. robusta* during LPS exposure in vivo remain unclear.

In the present study, using an in vivo LPS exposure strategy, next-generation sequencing (NGS) and qRT-PCR combined with bioinformatics and in silico analyses, we compared whole pharynx transcripts from naïve and LPS-exposed *C. robusta*. This combined approach provided the first indication of the involvement of the canonical Nf-κB pathway in inflammation, with the qRT-PCR analysis specifically suggesting interplay between Mif1 and Nf-κB signalling pathway components through the Tlr/MyD88-dependent pathway (including Tlr2, MyD88, and Irak4) during the biphasic activation of the inflammatory response upon LPS exposure. Furthermore, the presence of cis-acting GAIT elements in the 3′ untranslated region (UTR) of *Tlr2*, *MyD88*, *IκB*, *Ikkα*, and *Nf-κB* suggests the presence of both transcriptional and posttranscriptional control mechanisms involved in gene expression during the LPS-mediated inflammatory response of *C. robusta*.

## Results

### Transcriptomic analysis of *Ciona robusta* highlights the effects of LPS on transcripts involved in inflammation

**Figure 1:**
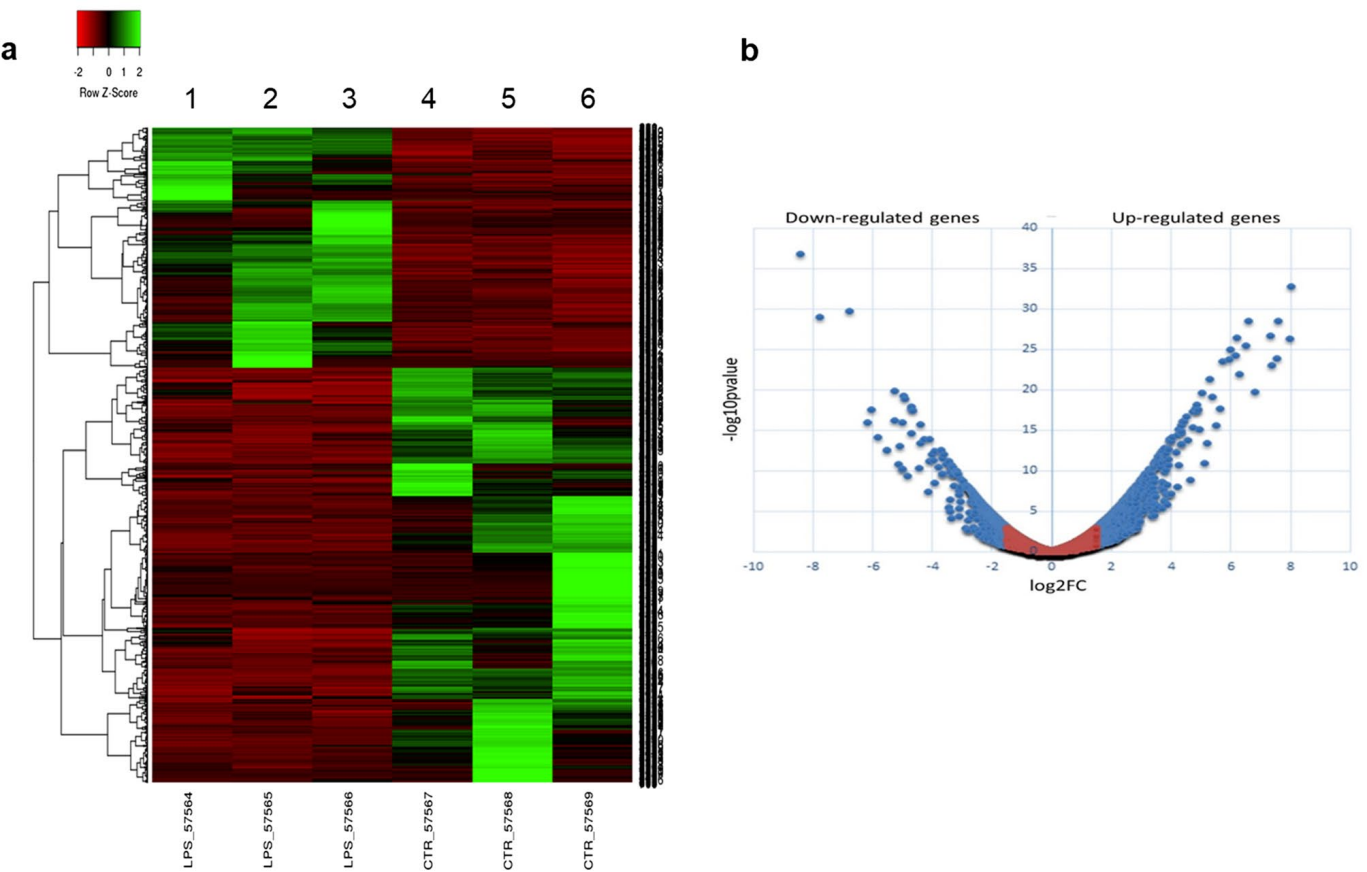
(**a**) Heatmaps of 1,227 differentially expressed unigenes (DEGs) in *C. robusta* after 4 h of LPS treatment. The figure shows LPS-treated (columns 1, 2 and 3) and untreated samples (columns 4, 5 and 6) in triplicate. (**b**) Volcano plot of differentially expressed DEGs in the *C. robusta* pharynx based on the comparison of the 4-h-LPS-treated to the untreated libraries. The expression level for each unigene is included in the plot. Blue points represent DEGs (up- and downregulated); red points represent non-DEGs. The y-axis indicates − log10 (p-value), and the x-axis indicates log2 (fold change). Genes significantly (p-value < 0.05) up- or downregulated (log2-fold change < 1.5 or log2-fold change > 1.5) are displayed in blue.

**Figure 2:**
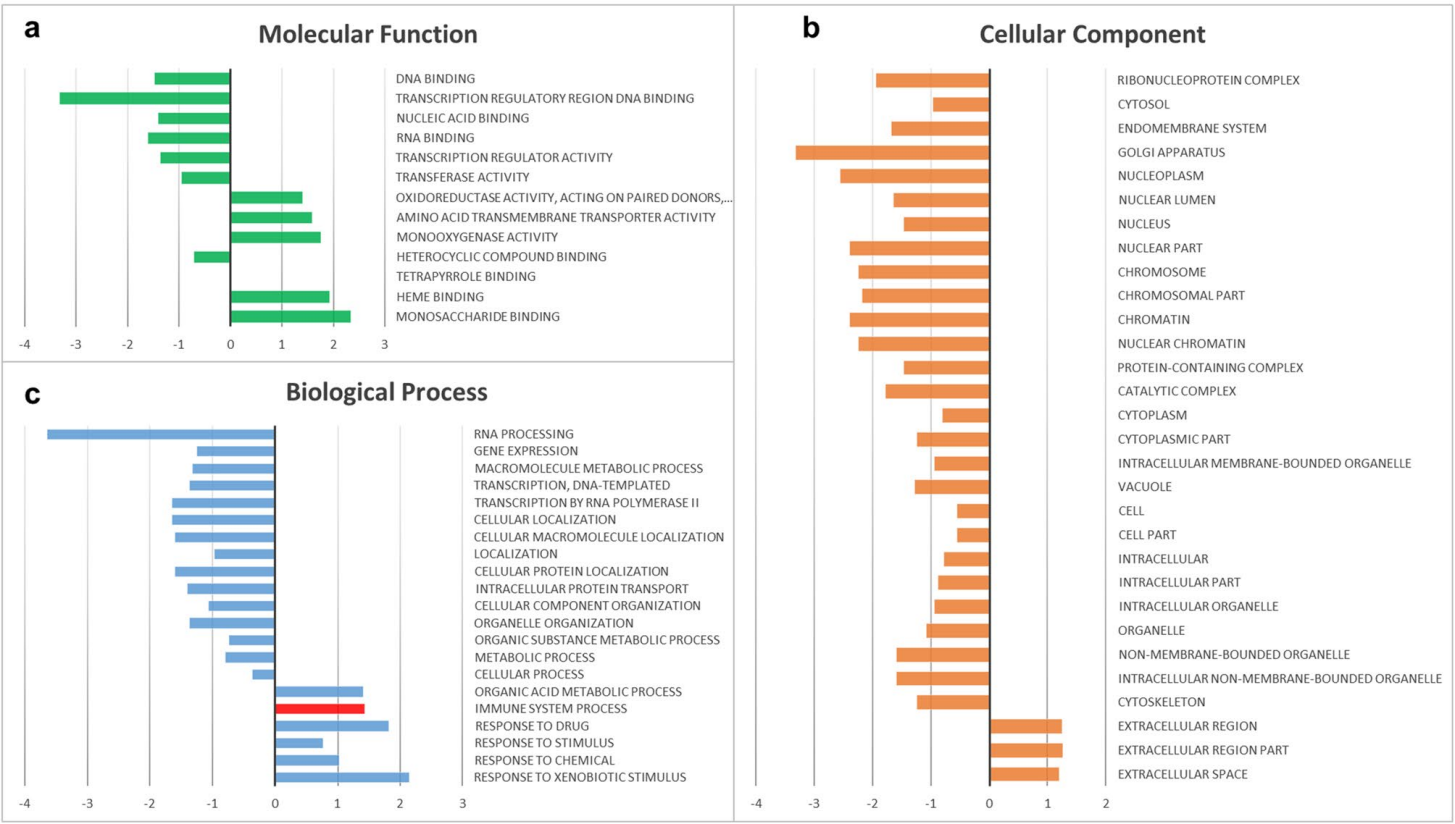
Gene enrichment analysis of differentially expressed genes, according to the three representative classes of GO: (**a**) molecular functions; (**b**) cellular components; (**c**) biological processes, for which the overrepresentation of immune system progress is shown in red. GO classes were filtered for p-value < 0.05 and FDR correction < 0.05 tests, according to the PANTHER GO-slim analysis tool.

To profile the *C. robusta* inflammatory response under LPS exposure in vivo, we investigated the differential gene expression in its immunocompetent organ (pharynx) under physiological and challenged conditions (4-h LPS exposure) using NGS transcriptomic analysis. Transcriptomic analysis under physiological conditions revealed 16,504 detected sequences, including protein-coding transcripts, non-protein-coding transcripts, isoforms, novel transcripts and protein-RNA interaction sites (data not shown). All 16,504 genes produced by sequencing were annotated using the Ensembl database (ensemble.org), and for each of them, log-fold changes, p-values and adjusted p-values were calculated (data not shown). With the log-fold change set at 1.5, 1,227 genes were found to be differentially expressed (p-value threshold < 0.05, and adjusted p-value < 0.05). A comparison between the basal gene expression of three specimens showed physiological inter-individual variability (Fig. 1a, compared with controls, columns 4, 5 and 6), but a large set of responsive genes (1,227) were found to be reproducibly regulated after 4 h of LPS exposure in vivo (p-value ≥ 1.5-fold higher or lower than controls) (Fig. 1a, columns 1, 2 and 3). Notably, the transcript levels of 447 target genes were found to be downregulated in response to LPS, whereas 780 genes were upregulated (Fig. 1b). For functional annotations, unigenes (1,227) were aligned to Gene Ontology (GO) terms based on the Protein ANalysis THrough Evolutionary Relationships (PANTHER) (pantherdb.org) classification system for gene ontology annotations, classified into three subcategories: (i) GO molecular functions (Fig. 2a); (ii) GO cellular components (Fig. 2b); (iii) GO biological processes (Fig. 2c).

**Figure 3:**
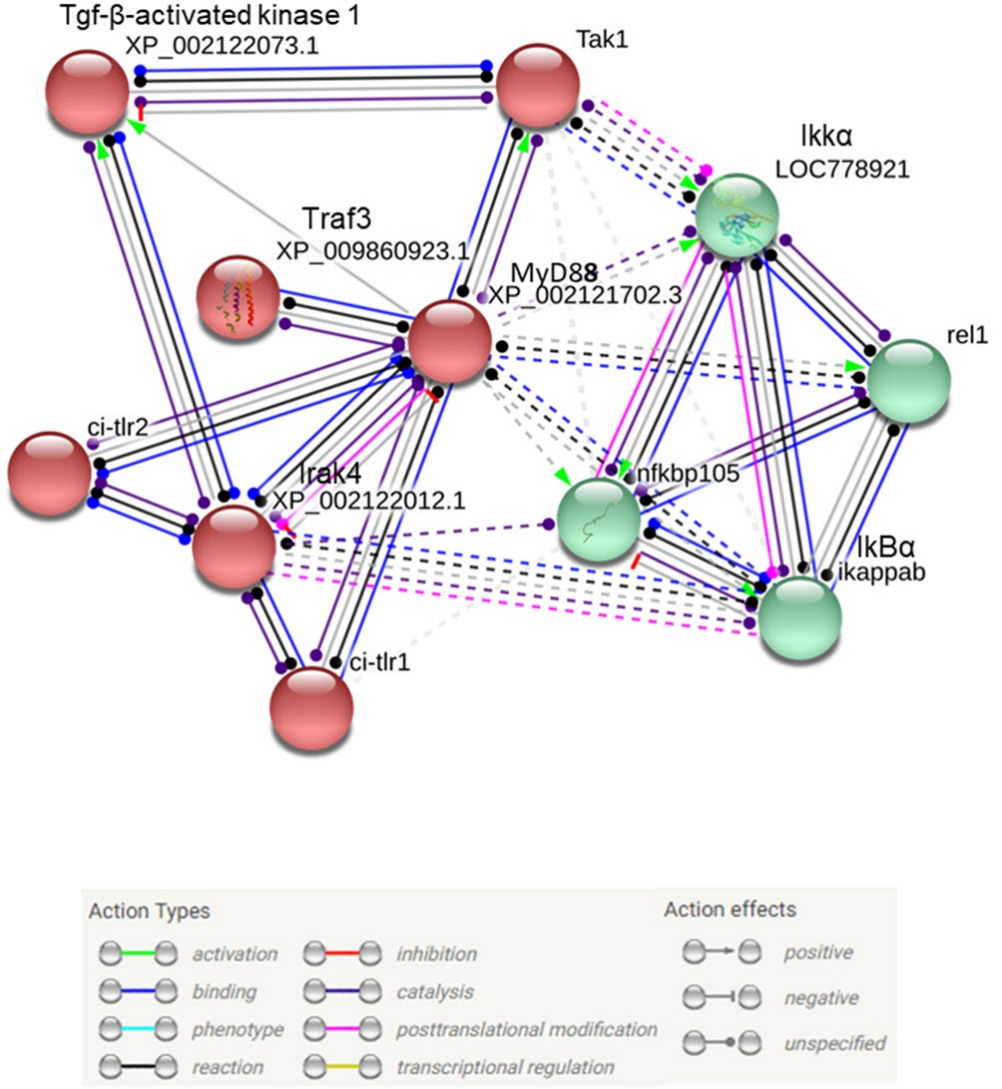
The Tlr-MyD88-dependent and Nf-κB pathways in *C. robusta*: protein–protein interaction network (PPIN) analysis of linked proteins by STRING. Nodes represent proteins, and edges represent direct and indirect interactions between proteins. Interesting edges are indicated by dashed lines. Clustering analysis was performed using the k-means algorithm, and two different clusters are identified in red and green. Edges represent predicted molecular action between network molecules (action types and effects are shown in the figure).

Genes responsive to LPS were found to be predominantly involved in molecular functions leading to gene expression and regulation (e.g., transcription regulatory region DNA binding, DNA binding, RNA binding), transporter activities (e.g., amino acid transmembrane transporter activity) and monosaccharide-binding activities (Fig. 2a). Gene expression programmes, which establish and maintain specific cell states, are controlled by transcription factors, cofactors, chromatin regulators and noncoding RNAs that interact with regulatory regions. Notably, genes involved in DNA-binding transcription activity were found to be significantly downregulated (3.5-fold decrease) upon LPS exposure, whereas those involved in amino acid transmembrane transporter and monosaccharide-binding activities were found to be upregulated (1.5- and 2.5-fold increase) (Fig. 2a). Concordantly, genes specific to cellular components were expressed at lower levels than the respective control group (e.g., processes involving the Golgi apparatus, nucleoplasm, chromosome, chromatin) (Fig. 2b). Within the biological processes category, the RNA-processing group was found to be strongly reduced (3.8-fold decrease) (Fig. 2c). On the other hand, the response to xenobiotic stimuli and drug processes was found to be moderately increased, whereas organic acid metabolic processes and the immune system processes were found to be slightly increased. The pathway analysis of differentially expressed unigenes with PANTHER revealed different pathways grouped into different categories (e.g., inflammation and immune response, growth factor/growth factor receptor-mediated pathways and intracellular signalling activation, apoptosis and p53 regulation, and metabolism). The first categories listed herein, according to GO analysis results, suggest a functional link among LPS inflammatory processes, cytokines, Tgf-β and Toll receptor signalling pathways (Table 1). STRING-protein–protein interaction networks functional analysis (string-db.org) was used to predict interactions among Tlr/MyD88-dependent signalling pathways and components of Nf-κB signalling by generating network clustered that are visualised according to the k-means algorithm (clustering analysis). The STRING analysis showed a greater functional link between Tlr2 and MyD88 (interaction score of 0.946) than was shown for Tlr1 and MyD88 (interaction score of 0.795) and suggested a strong functional link between Tlrs/Myd88 and Nf-κB, which was confirmed by the identification of two distinct functional clusters: the first included Tlr1, Tlr2, MyD88 and Irak4, and the second included Nf-κB, IκB, IKKα and Rel 1 (Fig. 3).

**Table 1:** Pathway analysis obtained with PANTHER tool: pathways were grouped in eight different categories, based on their potential relation.

Pathway id	Pathway name	Pathway category
P00031	Inflammation mediated by chemokine and cytokine signaling pathway	Inflammation and immune response
P00047	PDGF signaling pathway	
P00052	TGF-β signaling pathway	
P00054	Toll receptor signaling pathway	
P00057	Wnt signaling pathway	
P06211	BMP signaling pathway	
P06215	MYO signaling pathway	
P06217	*Drosophila melanogaster* Toll pathway	
P00053	T cell activation	
P00010	B cell activation	
P06959	CCKR signaling	Growth factors/growth factors receptors mediated pathways and intracellular signaling activation
P00012	Cadherin signaling pathway	
P00018	EGF receptor signaling pathway	
P00019	Endothelin signaling pathway	
P00034	Integrin signaling pathway	
P00021	FGF signaling pathway	
P00056	VEGF signaling pathway	
P04393	Ras Pathway	
P00032	Insulin/IGF pathway-mitogen activated protein kinase kinase/MAP kinase cascade	
P00032	Insulin/IGF pathway-protein kinase B signaling cascade	
P00016	Cytoskeletal regulation by Rho GTPase	Molecular switches
P00026	Heterotrimeric G-protein signaling pathway-Gi α and Gs alpha mediated pathway	
P00027	Heterotrimeric G-protein signaling pathway-Gq alpha and Go alpha mediated pathway	
P00028	Heterotrimeric G-protein signaling pathway-rod outer segment photo transduction	
P00005	Angiogenesis	Angiogenesis
P00011	Blood coagulation	
P00006	Apoptosis signaling pathway	Apoptosis and p53 regulation
P00059	p53 pathway	
P04397	p53 pathway feedback loops 2	
P05731	p53 pathway by glucose deprivation	
P00030	Hypoxia response via HIF activation	
P00014	Cholesterol biosynthesis	Metabolism
P00024	Glycolysis	
P00032	Insulin/IGFpathway-mitogen activated protein kinase kinase/MAP kinase cascade	
P00032	Insulin/IGFpathway-protein kinase B signaling cascade	
P00037	Ionotropic glutamate receptor pathway	
P00039	Metabotropic glutamate receptor group III pathway	
P02744	Fructose galactose metabolism	
P02730	Asparagine and aspartate biosynthesis	
P02762	Pentose phosphate pathway	
P00042	Muscarinic acetylcholine receptor 1 and 3 signaling pathway	Nervous system
P00043	Muscarinic acetylcholine receptor 2 and 4 signaling pathway	
P00044	Nicotinic acetylcholine receptor signaling pathway	
P00003	Alzheimer disease-amyloid secretase pathway	
P00004	Alzheimer disease-presenilin pathway	
P00007	Axon guidance mediated by semaphorins	
P00008	Axon guidance mediated by Slit/Robo	
P00009	Axon guidance mediated by netrin	
P00049	Parkinson disease	
P00029	Huntington	
P05731	GABA-B receptor II signaling	
P05912	Dopamine receptor mediated signaling pathway	
P00001	Adrenaline and noradrenaline biosynthesis	
P06212	DPP-SCW signaling pathway	*Drosophila melanogaste*r development related
P06213	DPP signaling pathway	
P06214	GBB signaling pathway	
P06216	SCW signaling pathway	
P00045	Notch signaling pathway

### Phylogenetic and in silico analyses of the Tlr/MyD88-dependent and Nf-κB canonical signalling pathways

**Figure 4:**
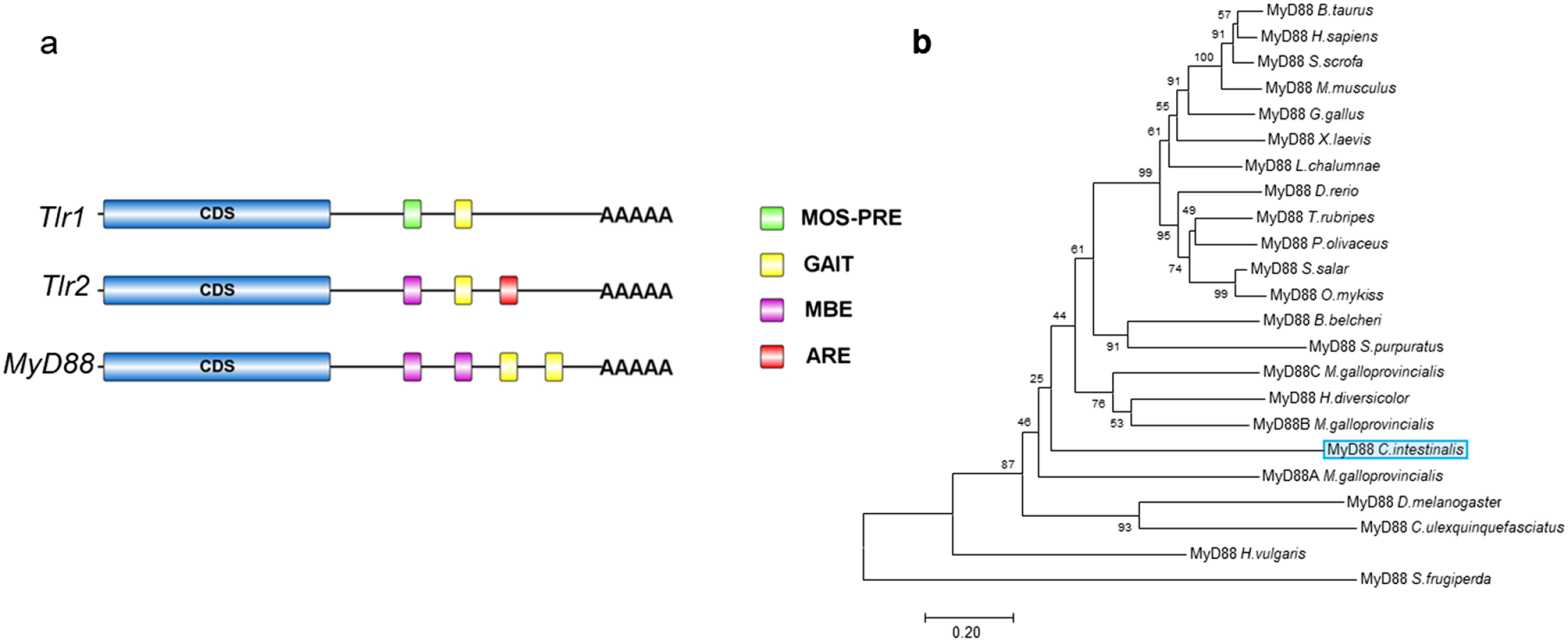
(**a**) Analysis of 3′-UTR mRNA sequences of *Tlr1*, *Tlr2* and *MyD88* using the RegRNA web tool: MOS-PRE (polyadenylation response element), GAIT (interferon-γ-activated inhibitor of translation), ARE (AU-rich element), MBE (Musashi-binding element); (**b**) phylogenetic tree of the MyD88 family of vertebrates and invertebrates. The tree was constructed using the neighbour-joining method and bootstrap analysis. The bootstrap value indicates the number of particular node occurrences per 1,000 trees, as generated by bootstrapping the sequences. The bar indicates the number of amino acid residue substitutions per site.

As previously noted*, C. robusta* possesses three TLR genes, and only two, called *Tlr1* and *Tlr2*, have been cloned^19^. Tlr proteins present the “hybrid” functionality of mammalian TLRs: Tlr1 (NP_001159599.2) shows 23% identity and 42% sequence similarity with the structurally and functionally well-characterised human TLR5, whereas Tlr2 (NP_001159600.1) shows 27% identity and 43% sequence similarity with human TLR8. In silico analysis was performed using the RegRNA2.0 database (https://regrna.mbc.nctu.edu.tw/html/prediction.html) to identify functional RNA motifs and elements in the 3′ (UTR) involved in *Tlr* mRNA post-transcriptional regulation. We found that the *Tlr1* 3′-UTR contains a γ-interferon activated inhibitor of translation (GAIT) element, a Mos polyadenylation response element (MOS-PRE) (Fig. 4a). GAIT elements are implicated in several immune-related mRNAs, showing important roles in gene-specific translation control in innate immunity; MOS-PRE is involved in both the translational repression of mRNA stored in immature oocytes and translational induction in maturing oocytes in *X. laevis;* in contrast, the *Tlr2* 3′-UTR contains a GAIT element, a Musashi-binding element (MBE), previously identified as being involved in mRNA translational control during cell cycle progression, and an AU rich element (ARE) (Fig. 4a).

The *C. robusta MyD88* gene has not yet been cloned, and to isolate full-length mRNA, we used a RACE 5′ and 3′ strategy. *MyD88* mRNA presents a 5′-UTR of 57 bp, an ORF of 1,210 bp and a 3′-UTR of 593 bp, which contains an MBE and GAIT element (Fig. 4a). It encodes a 403 amino acid polypeptide with a predicted molecular size of 45.169 kDa and a pI of 5.15 (Supplementary Fig. 1).

In silico analysis of the MyD88 amino acid sequence performed using both the Delta-Blast algorithm and the Simple Modular Architecture Research Tool (SMART) (smart.embl-heidelberg.de) jointly predicted the presence of specific structural domains: a DEATH domain (from 105 to 196 aa) and a Toll/interleukin-1 receptor (TIR) homology domain (from 265 to 399 aa) (Supplementary Fig. 1).

The *C. robusta* myD88-deduced amino acid sequences were examined in GenBank through Basic Local Alignment Search Tool (BLAST) (blast.ncbi.nlm.nih.gov) analysis and showed 28% identity and 48% sequence similarity with human MyD88. Molecular Evolutionary Genetics Analysis (MEGA X) software was used to build a phylogenetic tree by the neighbour-joining method (Fig. 4b). Phylogenetic analyses of vertebrate and invertebrate MyD88 proteins supported the idea of a conserved evolution from a common MyD88 ancestral gene among invertebrates, protochordates and vertebrates. The functionally and structurally conserved MyD88 motifs highlighted by CLC workbench 6.4 are shown in the supplementary material (Supplementary Fig. 2).

**Figure 5:**
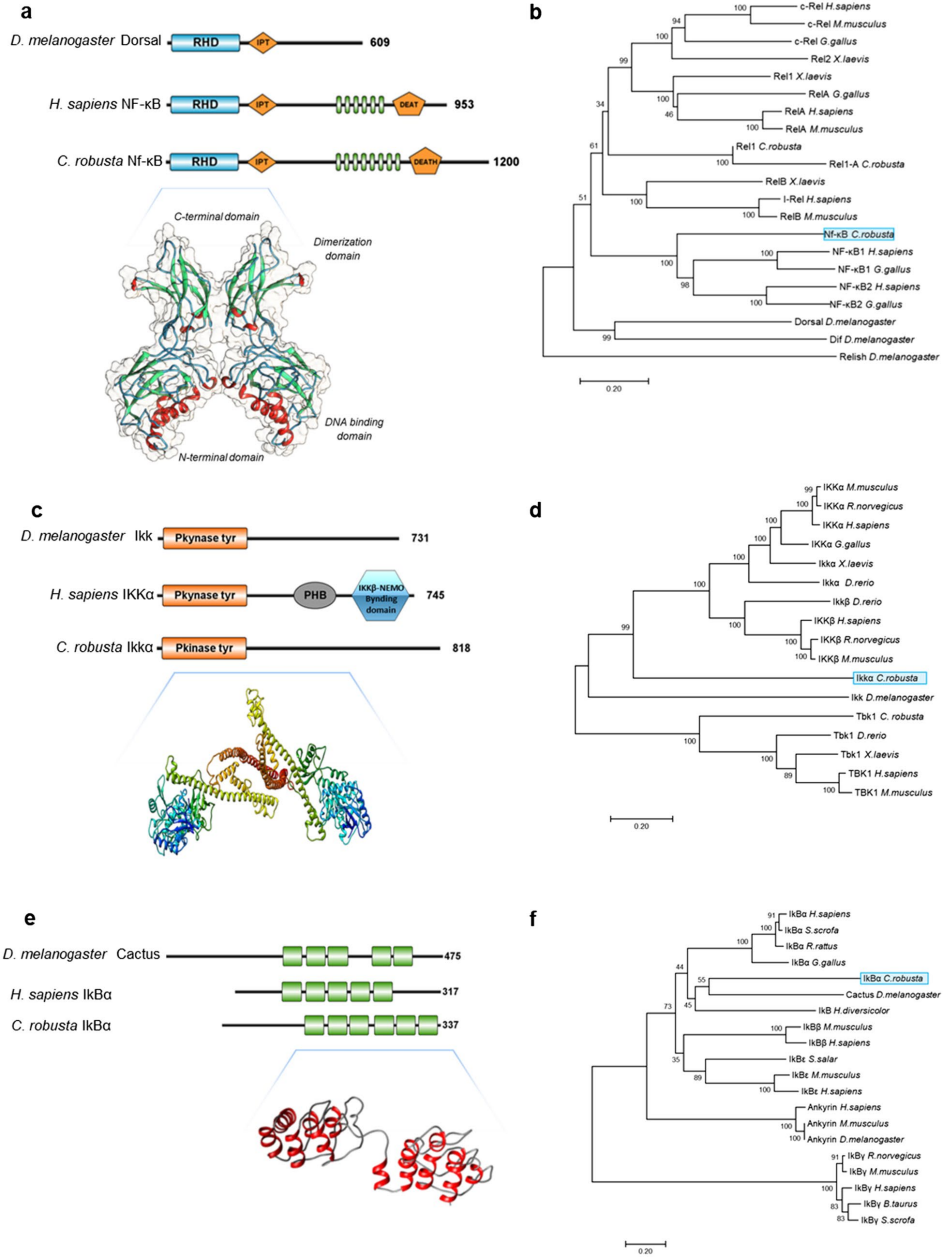
(**a**) Comparison of domain organisation in the *D. melanogaster* Dorsal, *H. sapiens* NF-κB1 and *C. robusta* Nf-κB proteins*.* The RHD domain is in blue, the ANK domain is in green, and the IPT and DEATH domains are in orange. Three-dimensional structures of *C. robusta* Nf-κB. (**b**) Phylogenetic tree of NF-κB and Rel family members in vertebrates and invertebrates. (**c**) Comparison of domain organisation in *D. melanogaster* Ikk, *C. robusta* Ikkα and *H. sapiens* IKKα. The Pkinase-Tyr domain is in orange, the PHB domain is in grey, and the IKKβ/NEMO-binding domain is in blue. Three-dimensional structures of *C. robusta* Ikkα. (**d**) Phylogenetic tree of IKK family members in vertebrates and invertebrates. (**e**) Comparison of domain organisation in the Cactus protein of *D. melanogaster*, *H. sapiens* and *C. robusta* IκBα. The ANK domain is in green. Three-dimensional structures of *C. robusta* IκBα. (**f**) Phylogenetic tree of IκB family members in vertebrates and invertebrates. The trees were constructed by the neighbour-joining method and bootstrap analysis. The bootstrap value indicates the number of particular node occurrences per 1,000 trees, as generated by bootstrapping the sequences. The bar indicates the number of amino acid residue substitutions per site. Nf-κB, Ikkα, and IkBα of *C. robusta* are highlighted in light blue boxes.

STRING-protein–protein interaction networks functional analysis suggested that in *C. robusta*, the major signalling pathways involved in the activation of Nf-κB were canonical pathways including Ikkα and IκBα proteins. In *C. robusta,* Nf-κB (NP_001071772.1) shows 53.36% identity with the structurally and functionally characterised NF-κB1 in humans and 41.49% identity with the Dorsal protein of the Rel subfamily present in *D. melanogaster*. In silico analysis using the SMART tool was performed to identify protein domains in *C. robusta* Nf-κB, *H. sapiens* NF-κB and *D. melanogaster* Dorsal proteins (Fig. 5a). For *C. robusta* Nf-κB, the search revealed a Rel homology domain (RHD) (26–215 aa), an IPT domain (222–324 aa), eight Ankyrin domains (797–1,046 aa) and a DEATH domain (1,083–1,170 aa), the same as *H. sapiens* NF-κB but with a different number of ANK domains (the tunicate Nf-κB has eight, and the human NF-κB has six); in *D. melanogaster* Dorsal, with a RHD and an IPT domain are present. Figure 5a shows the homodimer molecular model of *C. robusta* Nf-κB constructed according to the homology-modelling process performed on the basis of the known crystal structure of *H. sapiens* NF-κB1 (1svc.1.B) (Fig. 5a), generated from the super-imposition of the 2–326 residue sequence, shows 59.81% identity with the template. The phylogenetic tree representing the vertebrate and invertebrate NF-κB family members suggests that *C. robusta* Nf-κB is an orthologue of vertebrate *NF-κB* and that, in vertebrates, a single gene was duplicated into two (*NF-κB1* and *NF-κB2*) after the divergence of the tunicate and vertebrate lineages (Fig. 5b).

In humans, the activation of the NF-κB “canonical pathway” activates the protein kinase complex IKK^30,31^; in *C. robusta*, only one member of the Ikkα (XP_002125567.1) and IκBα (NP_001071739.1) families was identified. *C. robusta* Ikkα shows 31.53% identity with human IKKα and 32.62% identity with *D. melanogaster* Ikk. SMART analysis identified one PKinase-Tyr domain in *C. robusta* Ikkα (17–261 aa), *H. sapiens* IKKα and *D. melanogaster* Ikk (Fig. 5c)*.* Moreover, *H. sapiens* IKKα presents a PHB and an IKKα/NEMO-binding domain, both of which are involved in the formation of the IKK complex and are not present in *C. robusta* Ikkα or *D. melanogaster* Ikk. Figure 5c shows the homodimer molecular model of *C. robusta* Ikkα based on the known crystal structure of the *Mus musculus* serine/threonine-protein kinase (4jlc.1.A) generated from the super-imposition of the 12–79 residue sequence that shows 21.90% identity with the template. The phylogenetic analysis results support that supposition that *C. robusta Ikkα* is an orthologue of vertebrate *IKKα* genes (Fig. 5d). Finally, *C. robusta* IκBα (NP_001071739.1) shows 35.77% identity with *H. sapiens* IκBα and 31.60% identity with the Cactus protein of *D. melanogaster* family members and contains six ANK domains and tandem repeated modules of a 29-residue motif with a canonical helix-loop-helix-β-hairpin/loop folded structure (ANK1: 128–157; ANK2: 164–193; ANK3: 197–226; ANK4: 244–274; ANK5: 279–308; and ANK6: 312–341) (Fig. 5e), differing from *H. sapiens* IκBα and *D. melanogaster* Cactus, which each has five ANK domains. Figure 5e shows the homodimer molecular model of *C. robusta* IκBα based on the known crystal structure of *H. sapiens* Tankyrase-1 (3utm.1.A) generated from the super-imposition of the 123–367 residue sequence that shows 23.65% identity with the IkBα protein template. The phylogenetic analysis results support the supposition that *C. robusta IκBα* (Fig. 5f) is an orthologue of vertebrate *IκBα* genes and has amino-terminal signal-responsive regions containing conserved serine residues (e.g., Ser^84^ and Ser^88^) that are phosphorylated in an 83-DSPGXXSP-89 amino acid sequence. An in silico analysis was performed based on RegRNA2.0 databases to identify functional RNA motifs and elements in the 3′-UTR of the *Nf-κB, Ikkα* and *IκBα* genes with identified GAIT elements (data not shown).

### Analyses of the expression of *Tlr/MyD88*-dependent and *Nf-κB* canonical signalling pathways and immune-related genes under LPS exposure

**Figure 6:**
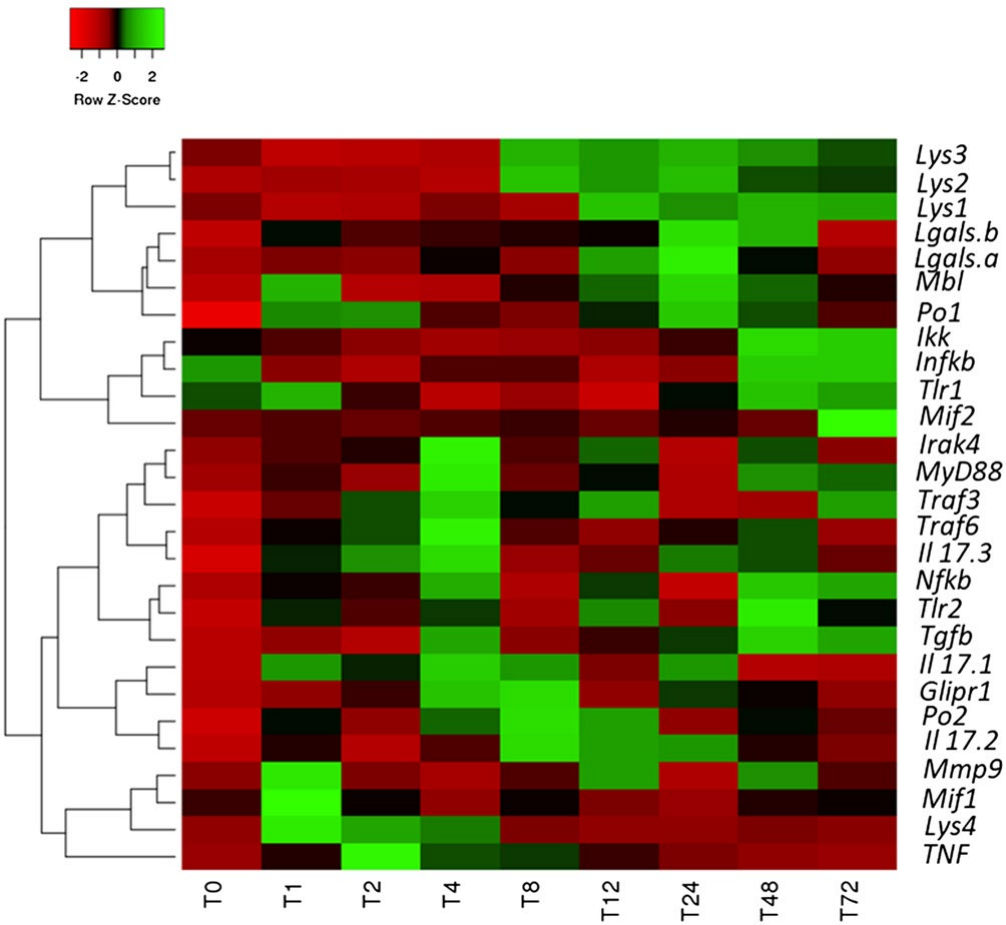
Heatmap based on the qRT-PCR analysis of the differentially expressed Tlr/MyD88-dependent and Nf-κB pathway and immuno-related genes at different times of exposure to LPS (1–72 h). Time course of gene expression in the pharynx of *C. robusta* exposed to LPS compared with the gene expression in untreated ascidians. Values are presented as the means ± SD. Statistical differences were estimated by one-way ANOVA and Tukey's t-test. The level of significance was set at a p-value ≤ 0.05 (N = 4).

Analyses of the time-course expression of *Tlr/MyD88*-dependent and *Nf-κB* canonical signalling pathway immune-related genes in the pharynx inflammatory response induced by LPS in *C. robusta* were performed at time points from 0 to 72 h post-LPS challenge by qRT-PCR (Fig. 6). The heatmap shows that a large portion of the transcripts was significantly modulated in response to LPS during the 72-h period of LPS exposure (p-value < 0.05). Based on the expression patterns of the transcripts, two major clusters were highlighted: the first includes proinflammatory cytokines and components of *MyD88/Nf-κB* signalling, and the second comprises the *Mif2* cytokine *IκBα* and a few inflammation effectors (e.g., lectins, lysozymes, and Po). Specifically, the heatmap highlighted that the inflammatory cytokines *Mif1*, *Il-17–1*, *Il-17–3*, and *Tnf-α* and the metalloprotease *Mmp9* were upregulated between 1 and 4 h of LPS exposure (p-value < 0.05). *Tnf-α* reached its maximum expression level after 2 h of LPS exposure. Notably, *Tlr2*, *MyD88*, *Irak4*, *Nf-κB* and transforming growth factor β (*Tgf-β*) transcripts displayed a significant increase after 4 h of LPS exposure (p-value < 0.05). On the other hand, after 8 h of exposure, *Il-17–1*, *Il-17–2,* and *Mmp9* levels began to increase s they had at 1 h. *Tlr2*, *MyD88*, *Irak4*, *Nf-κB* and *Tgf-β* experienced a second significant increase after 48 h of exposure (p-value < 0.05). Our findings suggest biphasic activation of the inflammatory response upon LPS exposure, with a first wave of activation from 0 to 12 h and a second wave of activation from 12 to 72 h.

## Discussion

In this study, we found that the innate immune signalling pathway activated by LPS in *C. robusta* is evolutionarily conserved and involves both the Tlr and Mif signalling pathways, findings in agreement with reports highlighting the key immune signalling pathways activated against invading microbial pathogens and other potential threats to a mammalian host^46^. In general, these findings are not surprising, since this invertebrate occupies a key phylogenetic position in chordate evolution and is considered to be a member of the sister group of vertebrates^5–7^. In our model, the Mif signalling pathway seems to be activated as the first line of defence against the bacterial endotoxin LPS and appears to be a major regulator of *Il-17s*, *Tgf-β* and *Mmp9* transcription, as these levels were found to be upregulated after 1 h of exposure. In contrast, the Nf-κB signalling pathway seems to be recruited a few hours later to activate Tgf-β through the involvement of Tlr2, MyD88 and Irak4.

In humans, LPS induces TLR4 activation through binding with three proteins (the lipopolysaccharide-binding protein LBP, the cluster differentiation antigen CD14, and the myeloid differentiation protein receptor MD-2)^47^. In humans, MD-2 is a component of the MD-2-related lipid-recognition (ML) superfamily, which contains a large set of genes encoding proteins such as MD-1, MD-2, and Niemann-Pick type C2 (NPC2) protein. The ascidian *C. robusta* possesses two Tlr-related genes presenting “hybrid” biological and immunological functionality of mammalian TLRs^19^, but LPS significantly activates only one of them, Tlr2; on the other hand, no MD-2 orthologue was found in the genome of *C. robusta*. The subtractive hybridization strategy allowed the identification of *Npc2* mRNA in *C. robusta,* an homologue of ML superfamily components, preferentially expressed in haemocytes inside the vessel lumen of the pharynx^48^. This finding supports the hypothesis that the recognition of LPS by TLR4 through MD-2 binding may have been gained early during the evolution of vertebrates and that *C. robusta* may respond to LPS through a complex with alternative LPS sensors (i.e., the Ncp2 protein) developed before the differentiation of the MD-2 protein in vertebrates. LPS activation of Tlr2 likely regulates the Rel/Nf-κB signal transduction pathway, and we hypothesise that Tlr1 in is involved in the signalling that activates Mif1 in *C. robusta*. Notably, the molecular mechanisms underlying Tlr ligand recognition and signal transduction are distinct from those of vertebrate TLRs^19^. The qRT-PCR results suggest an intriguing possibility of biphasic action of the Nf-κB and Mif signalling pathways during the 72 h of exposure to LPS, suggesting their different roles in regulating immune cell behaviour, respectively, with a pro-inflammatory action period (from 0 to 12 h) and an anti-inflammatory action period at which time homeostasis is restored (from 12 to 72 h).

Recently, a few studies have indicated that, in humans, intracellular MIF is involved in the induction of NF-κB activity^49^, reinforcing the idea of a close relationship between Mifs, Tlrs and Nf-κB in *C. robusta*. In agreement, Roger et al.^50^ found that MIF upregulates *Tlr4* expression in mouse macrophages and activates the MAPK and NF-κB signalling pathways in immortalised cell lines and mouse macrophages^46^. In humans, the upregulation of *MIF* during inflammation and under other stress conditions is primarily regulated at the level of MIF release rather than transcriptional induction^51^, which explains its rapid activation. In *C. robusta*, *Mif2* is not regulated by LPS, and its 3′-UTR mRNA has a CPE element, confirming that it can be stored in the cytoplasm for rapid activation by a cytoplasmic polyadenylation mechanism; instead, *Mif1* is upregulated after LPS stimulation and does not have a 3′-UTR CPE^45^. Moreover, the in silico analysis of the 3′-UTR of *Tlr1* revealed a MOS-PRE that includes the 5′ sequence of the CPE^52^. Both the CPE and MOS-PRE enable translational repression or activation in response to specific stimuli; therefore, we can hypothesise that, following LPS stimulation, *Mif1* and *Tlr1* transcripts gain poly(A) tails, which activate their translation and allow the rapid and abundant expression of proteins for their prompt response.

In humans, MIF can regulate TNF and other cytokines by affecting the expression of *TLR4*, *p53*, *ERK*, *c-Jun-N-terminal kinase*, *p38*, and *MAPK phosphatase-1*^53^. On the other hand, the stimulation of dendritic cells with TLR4 ligands elicits high levels of MIF production, and secreted MIF acts as an autocrine/paracrine enhancer of TNF production. Our findings support the notion that there is interplay between Mifs and Nf-κB pathway components in *C. robusta*, meaning that they have an effect on each other. Specifically, we speculate that to actively maintain the immune response and increase its efficiency, Mif2 and Tlr1 are involved in a rapid response (within a few minutes) and stimulate the transcription of *Mmp9* and the pro-inflammatory cytokines *Mif1*, *Tgf-β*, *Il-17* and *Tnf-α,* which, in turn, activate *Nf-κB* through Tlr2, eliciting renewed *Mif2* activation until immune response resolution. In silico analyses of the cis-regulatory elements of the 3′-UTR in *C. robusta* based on RegRNA 2.0 revealed a GAIT element in *Tlr2*, *MyD88*, *IκB*, *Ikkα*, and *Nf-κB*. In humans, GAIT elements are implicated in several immune-related mRNAs, showing an important role in gene-specific translation control in innate immunity^54^. In *C. robusta*, GAIT elements have already been identified in *Cap*^55^ and *Tnf-α*^56^ and in *Mif* mRNAs^45^. Notably, in *C. robusta,* Mifs also seem to regulate *Mmp9*, which has several important immune functions, such as extracellular matrix degradation and Tnf-α and Tgf-β activation^57^. In humans, TGF-β is a crucial enforcer of immune homeostasis and tolerance, inhibiting the expansion and function of many immune system components. It can be viewed as immunosuppressive and plays important roles both during the initial phase of immune injury and during the later remodelling phase^58^. In agreement with these findings, *Tgf-β* was overexpressed in *C. robusta* after 4 h of LPS exposure and later, from 12 to 72 h, thus regulating the resolution of the inflammatory response.

**Figure 7:**
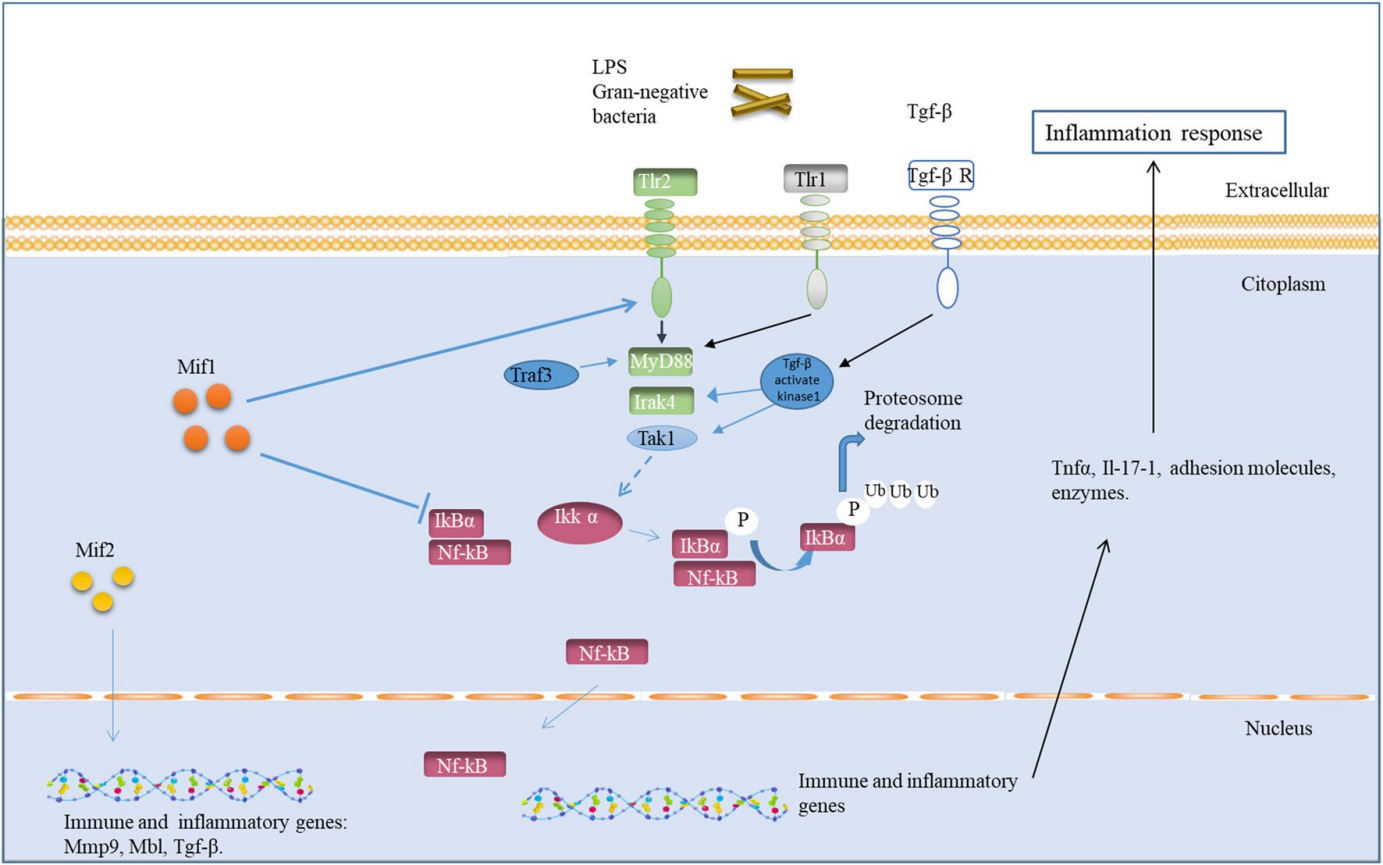
Schematic model representing the putative interplay between the Mif and Nf-κB pathways in *C. robusta*. After an external stimulus such as LPS treatment, Tlrs activated intracellular signalling such as that by MyD88 and Irak4 proteins. Mif1 is involved in the activation of the *Nf-κB* transcription factor through the downregulation of the inhibitory IκB protein and the upregulation of *Tlr2*. In the cytoplasm, the signalling cascade activated by MyD88 causes Nf-κB activation through Ikk molecules. IκBα is then ubiquitinated, and Nf-κB is translocated to the nucleus, where it activates mRNA transcription of inflammatory genes. Cytokines, metalloproteases, adhesion molecules and other inflammatory proteins are then produced, activating the inflammatory response.

Finally, we propose a novel schematic model representing the putative interplay between the Mif and Nf-κB pathway components (Fig. 7). To the best of our knowledge, this is the first study to show evidence of interplay between the Mif and Nf-κB pathways using a marine invertebrate model through a wide-ranging approach. Further studies in this direction are needed to cover knowledge gaps regarding the hierarchically organised set of molecular, cellular and organismal networks involved in universal immune interactions with pathogens and subsequent intracellular signal transduction.

## Methods

### Tunicates and LPS injection

Molecular studies have led to the hypothesis that *Ciona intestinalis* constitutes a compilation of species rather than a single species^10–13^. Accordingly, our model organism, originally from the Mediterranean Sea and formerly classified as *C. intestinalis*, corresponds to *C. robusta*^10–13^.

*C. robusta* specimens were collected from Sciacca Harbour (Sicily, Italy) and were acclimatised and maintained under controlled temperature conditions (15 °C) in tanks supplied with flow-through oxygenated seawater. The animals were fed every 2 days with Coraliquid marine invertebrate food (Sera Heinsberg, Germany). An LPS solution (*Escherichia coli* 055:B5, LPS, SIGMA-ALDRICH, Germany) was prepared in sterile salt medium (12 mM CaCl_2_, 11 mM KCl, 26 mM MgCl_2_, 43 mM Tris HCl, 0.4 M NaCl, pH 8.0). One hundred microlitres of the LPS-containing suspension was injected into the tunic matrix surrounding the pharynx wall (median body region) at a final LPS concentration of 100 μg. *C. robusta* not exposed to LPS (*naïve*) were used as controls. Fragments of pharynx tissue (200 mg) explanted at various times (from 1 to 72 h) were immediately soaked in RNAlater tissue collection solution (AMBION, Austin, TX) and stored at − 80 °C. Total RNA extraction was performed using an RNAqueous-Midi kit purification system (AMBION, Austin, TX).

### Transcriptomics

The RNA purity and quality of total RNA extracted from the pharynx of *C. robusta* that were *naïve* (N = 3) and that were exposed to LPS for 4 h (N = 3) were examined by NanoDrop and Agilent RNA 6,000 Nano kits on an Agilent 2,100 Bioanalyzer (AGILENT, USA), respectively. High-quality RNA samples (A260/A280 = 1.9–2.1, RIN ≥ 7) were used for cDNA library construction. RNA sequencing (RNA-Seq) was performed by BMR Genomics (Padua, Italy) on an Illumina platform in a single-end format 75 bp (1 × 75 bp) containing ~ 40 million ± 10% of reads/sample. An analysis of differentially expressed genes was performed by BMR Genomics (Padua, Italy) using edgeR software, which estimates the negative binomial variance parameter globally across all genes. All data from the edgeR analysis were normalised by setting the false discovery rate (FDR) to ≤ 0.05 and the absolute value of the log fold change (logFC) to 1.5. A heatmap and a volcano plot were generated to visualise the results indicating the genes differentially expressed between the exposed samples and controls. The heatmap was produced using a heatmapper tool (https://www.heatmapper.ca). Complete linkage clustering was applied, and Spearman’s rank correlation was used as the method of distance measurement. In the volcano plot, the log_2_CPM, which represents the log base 2 of the count per million (CPM) for each transcript, was calculated by the CPM function. Negative log values represent transcripts presenting a CPM lower than 1, and positive log values represent those presenting a CPM ≥ 1.

### Gene functional enrichment analysis and immune network construction

The Clusters of Orthologous Genes (COG) database (geneontology.org) was used to perform a Gene Ontology (GO) enrichment analysis of a set of *C. robusta* transcripts produced by NGS. The ontology analysis was divided into three subcategories: (i) molecular functions (MF); (ii) cellular components (CC); (iii) and biological pathways (BP). The Protein Analysis Through Evolutionary Relationships (PANTHER GO-slim analysis tool) System connected to the COG database was utilised to expand the annotation data of the gene and protein families obtained from GO. A GO analysis was performed by selecting the PANTHER “GO-slim” analysis mode, as it is more reliable and accurate than the GO annotation mode “GO-complete”. Different parameters used to measure the functional role of the list of genes were taken into account and reported as fold enrichment. In addition to p-value evaluation (p-value < 0.05), false discovery rate (FDR < 0.05) control procedures were also considered. The COG database was also used to perform pathway analysis of the NGS data. The Search Tool for the Retrieval of Interacting Genes/Protein (STRING) database, which allows the visualisation of complex networks (through clustering analysis), was used to retrieve the predicted interactions for the identified immune-related proteins. Levels of significance were set at a p-value < 0.05; the log2-fold change was </> 1.5. Significant data (proteins significantly modulated) were clustered according to the k-means algorithm, an unsupervised clustering algorithm based on an adjacency matrix.

### Cloning and bioinformatics analyses of *MyD88*

The Ensembl automatic annotation of the genome sequence (https://www.ensembl.org/) was used to identify the following sequence: *MyD88* (ENSCING00000017616). *MyD88* cDNA was obtained using the GeneRacer kit (INVITROGEN, USA), as reported in Vizzini et al.^45^. In short, 5′- and 3′-RACE was directed by the listed primers reported in supplementary Table 1; the overlapping RACE was cloned into a pCRII vector (TA cloning kit, INVITROGEN, USA) and sequenced. cDNA was amplified by polymerase chain reaction (PCR), and the amplicon was cloned into a pCRII vector (TA cloning kit, INVITROGEN, USA) and sequenced. The full-length *MyD88* cDNA was analysed with the ExPASy translation tool (https://web.expasy.org/translate/) to obtain the open reading frame (ORF) regions, the leader and trailer sequences (UTRs) and the nucleotide sequence, which was translated into a protein sequence. The protein sequence was located using the Basic Local Alignment Search Tool (BLAST, NCBI) to identify known protein sequences that are homologous to MyD88, and the physical/chemical parameters (e.g., molecular mass and isoelectric point) were estimated by the Prot-Param tool in ExPASy (https://www.expasy.org/tool/protparam/). The NCBI Conserved Domain database (https://www.ncbi.nlm.nih.gov/Structure/cdd/wrpsb.cgi) was used to predict the domain architecture, and other conserved domains and functional motifs were determined by the PROSITE database (https://prosite.expasy.org/scanprosite/). The SignalP 4.0 server (https://www.cbs.dtu.dk/services/SignalP/) was used to predict the putative cleavage site of the signal peptide, and CLC Genomics Workbench software (Version 7.0.0) was used for the multiple sequence alignment.

### Structural and phylogenetic analyses

As reported in Vizzini et al.^45^, secondary structures were evaluated using Polyview software (https://polyview.cchmc.org), and 3D structures were predicted using Phyre2 (Protein Homology/analogY Recognition Engine V 2.0; https://www.sbg.bio.ic.ac.uk/phyre2/html/page.cgi?id=index). Structures were validated by Ramachandran plot analysis (https://mordred.bioc.cam.ac.uk/∼rapper/rampage.php). Homology modelling was performed on the basis of the known crystal structures. Phylogenetic trees were designed in MEGA X maintaining the bootstrap value of 1,000 bootstrap iterations (neighbour-joining method). The accession numbers are listed in Table 2. The Regulatory RNA Motifs and Elements Finder tool (https://regrna.mbc.nctu.edu.tw/html/prediction.html) was used to characterise the 3′-UTRs.

**Table 2:** Accession numbers.

Name	GenBank no.
*Salmo salar* MyD88	ABV59003.1
*Oncorhynchus mykiss* MyD88	NP_001117893.1
*Xenopus laevis* MyD88	NP_001081001.1
*Paralichthys olivaceus* MyD88	XP_019943061.1
*Branchiostoma belcheri* MyD88	XP_019623569.1
*Gallus gallus* MyD88	NP_001292020.1
*Latimeria chalumnae* MyD88	XP_005994282.1
*Sus scrofa* MyD88	NP_001093393.1
*Danio rerio* MyD88	NP_997979.2
*Bos taurus* MyD88	NP_001014404.1
*Homo sapiens* MyD88	AAC50954.1
*Mus musculus* MyD88	AAC53013.1
*Drosophila melanogaster* MyD88	NP_610479.1
*Haliotis diversicolor* MyD88	AHK60398.1
*Mytilus galloprovincialis* MyD88c	AGG10812.1
*Mytilus galloprovincialis* MyD88a	AFR54116.1
*Mytilus galloprovincialis* MyD88b	AGG10811.1
*Amphimedon queenslandica* MyD88	ADR78337.1
*Culex quinquefasciatus* MyD88	EDS31829.1
*Hydra vulgaris* MyD88	CDG67778.1
*Spodoptera frugiperda* MyD88	AFK24444.1
*Ciona* IKKα	NP_001071740.1
*Homo sapiens* IKKα	NP_001269.3
*Mus musculus* IKKα	NP_031726.2
*Danio rerio* Ikkα	NP_956611.1
*Xenopus laevis* Ikkα	NP_001086127.1
*Rattus norvegicus* IKKα	NP_001101058.1
*Gallus gallus* IKKα	NP_001012922.1
*Rattus norvegicus* IKKβ	NP_445807.2
*Mus musculus* IKKβ	NP_001153246.1
*Danio rerio* Ikkβ	NP_001116737.1
*Homo sapiens* IKKβ	NP_001547.1
*Homo sapiens* TBK1	NP_037386.1
*Mus musculus* TBK1	NP_062760.3
*Danio rerio* Tbk1	NP_001038213.2
*Xenopus laevis* Tbk1	NP_001086516.1
*Ciona* Tbk1	XP_002125567.1
*Homo sapiens* IkBα	NP_065390.1
*Sus scrofa* IkBα	CAA84619.1
*Rattus norvegicus* IkBα	Q63746.1
*Gallus gallus* IkBα	Q91974
*Drosophila melanogaster* Cactus	AAA85908.1
*Mus musculus* IkBβ	NP_001293151.1
*Homo sapiens* IkBβ	AAD08997.1
*Rattus norvegicus* IkBγ	Q6TMG5.1
*Bos taurus* IkBγ	CAC93688.1
*Homo sapiens* IkBγ	AAC36330.1
*Mus musculus* IkBγ	O88522.2
*Sus scrofa* IkBγ	A9QT41.1
*Mus musculus* IkBε	NP_032716.2
*Homo sapiens* IkBε	NP_004547.3
*Salmo salar* IkBε	ACN11190.1
*Haliotis diversicolor* IkB	AHM27300
*Ciona robusta* IkB	NP_001071739.1
*Homo sapiens* Ankyrin	AAA51732.1
*Mus musculus* Ankyrin	AAA37236.1
*Drosophila melanogaster* Ankyrin	AAC37208.1
*Homo sapiens* c-Rel	CAA52954.1
*Mus musculus* c-Rel	CAA42817.1
*Gallus gallus* c-Rel	NP_001161198.1
*Xenopus laevis* Rel2	NP_001079306.1
*Homo sapiens* RelA	AAH33522.1
*Mus musculus* RelA	AAF82405.1
*Xenopus laevis* Rel1	Q04865.1
*Gallus gallus* RelA	XP_025001046.1
*Homo sapiens* I-Rel	AAA36127.1
*Mus musculus* RelB	NP_033072.2
*Xenopus laevis* RelB	NP_001079335.1
*Drosophila melanogaster* Dorsal	NP_724052.1
*Drosophila melanogaster* Dif	AAA28465.1
*Homo sapiens* NF-kB1	CAB94757.1
*Gallus gallus* NF-kB1	XP_015140904.1
*Homo sapiens* NF-kB2	NP_001309863.1
*Gallus gallus* NF-kB2	NP_989744.1
*Drosophila melanogaster* Relish	NP_477094.1
*Ciona robusta* Nf-kB1	Q4H348
*Ciona robusta* Rel1	NP_001029013.1
*Ciona robusta* Rel1-A	XP_026692312.1

### qRT-PCR

The differential expression of 27 LPS immune responsive genes was studied by qRT-PCR using the SYBR-Green method and the specific sets of primers listed in Table 3. qRT-PCR analysis was performed using an APPLIED BIOSYSTEMS 7500 Real-time PCR system^45^. Differential expression was determine in a 25 µl PCR mixture containing 2 μl of cDNA converted from 250 ng of total RNA, 300 nM primer (forward and reverse), and 12.5 μl of Power SYBR-Green PCR MasterMix (APPLIED BIOSYSTEMS). Amplification specificity was tested by real-time PCR melting analysis. To obtain sample quantification, the 2^−ΔΔCt^ method was used, and the relative changes in gene expression were analysed as described in the APPLIED BIOSYSTEMS Use Bulletin N.2 (P/N 4303859). The transcript levels from different tissues were normalised to that of actin to compensate for variations in the amount of RNA input. Relative expression was determined by dividing the normalised value of the target gene in each tissue by the normalised value obtained from the untreated tissue. To examine the time course of the response, LPS-treated ascidians (N = 4) were examined at incremental post-inoculation time points (1, 2, 4, 8, 12, 24, 48, and 72 h). Untreated ascidians (*naïve*) (N = 4) were used as controls.

**Table 3:** Primers used for qRT-PCR.

Gene	Primer sequence (5′–3′)
*Mif1*	5′-GCTTGCAGCGCTTTTGATG-3′ 5′-AAACGGGTTCCAGAAACTCCTAA-3′
*Mif2*	5′-CCATGAAGCAACGAGGGAAA-3′ 5′-TTCTTGGCTGCGAGTTGGT-3′
*Lys-g1*	5′-AACTTTGTATGGACGCTGCTG-3′ 5′-GCCCCTGCACGACTTTCA-3′
*Lys-g2*	5′-CACGGTGCCGACACAAAGT-3′ 5′-GCGCCTTGTAAAATGTGATCTC-3′
*Lys-g3*	5′-GCAAGCCGCGAAAGCA-3′ 5′-TCACCAAGCCCGTCTTTGTC-3′
*Lys-g4*	5′-CGGCGTAGCCATCGCTTA-3′ 5′-CGGTGGTGTCAGTGTTGTAGAT-3′
*Gal-a*	5′-TGTTGAATGGCTTCCACTTGTT-3′ 5′-TCGGATGTTACGCAGAGGTTT-3′
*Gal-b*	5′-CTCGGTGTATGTGAACGATGTTC-3′ 5′-CGGTAGAGCGGCACCTTGT-3′
*Mbl*	5′-AGCCTTGACGTTCGCAGAGT-3′ 5′-AGCCTTGACGTTCGCAGAGT-3′
*Po1*	5′-ATACCCGGACAAGATCACCATG-3′ 5′-TGGAGAGGTTCTCAGCTGCTTC-3′
*Po2*	5′-CCCCTATTAGAGTGAATGGCCA-3′ 5′-CAAAGAGATCCACTGGTGCAGA-3′
*Glipr1*	5′- GTATCTCCGCGAAGAGTTGG-3′ 5′-TCGGTATAACGTCGCCTCT-3′
*Mmp9*	5′- GACGAGTTCCGCGTAACGTT-3′ 5′- ATGGAATACCGTGCTCTTGTAGGA-3′
*Tgf-β*	5′-TTTCAGGGACCCAAAAACGA-3′ 5′-GCCAGCTATAATGACATCCAAGGT-3′
*Tnf-α*	5′-GCCTCCCATAGACCGTTGTTAA-3′ 5′-CGGGACACCTTCAGCACAT-3′
*Il-17–1*	5′- GCCGGGAACGTGACAGAA-3′ 5′-GGCATGTTGATTGCGACCTT-3′
*Il-17–2*	5′-GTGTAGCGGGTGCATTGCT-3′ 5′-GGCACCGACTTCCCAACA-3′
*Il-17–3*	5′-CAAAGCGGAGCCTTCAATGT-3′ 5′-GCTTCTTTGCTCGACACTTGTG-3′
*Tlr1*	5′-GCTATCGAGAACCCGCCATA-3′ 5′-AATCACGGGAAGGAAAGCAA-3′
*Tlr2*	5′-CCTGCTTCAAAACCTCCAATCT-3′ 5′-TTTAAGGAAAACGGGTTATTGTACTATG-3′
*MyD88*	5′-TCGGAATGCCTAGAAGTGATTTCT-3′ 5′-TGCGTGAGTCGAAACGTAGATG-3′
*Irak4*	5′-TCCCCCATACACCCTGTACCT-3′ 5′-GCGACTTAAATCCAAGCGAAA-3′
*Traf3*	5′-ACGGGTGAGGTCCGTTAGG-3′ 5′-TGCGGTAATGGGCCAAGT-3′
*Traf6*	5′-TAAACCGCATCCCGGTACTT-3′ 5′-GCGGGTTTTATCCAAATTTACG-3′
*IkBα*	5′-TTATGTGGCACTTGGTATGGAGTATT-3′ 5′-CCGGCTGAGTCAGGAACTTC-3′
*Ikkα*	5′-AGGCGGTGGCTGAACTGA-3′ 5′-TTCCCCATCGAACAGAAGAAGT-3′
*Nf-kB*	5′-GCCGACGTACTGCTTTGCA-3′ 5′-GCCAGCCACCACGATGTT-3′
*Actin*	5′-TGATGTTGCCGCACTCGTA-3′ 5′-TCGACAATGGATCCGGT-3′

### Statistical methods

Statistical assessments of GO term enrichment and pathway analyses were performed by Fisher's exact test in combination with a robust false discovery rate (FDR) correction for multiple testing.

The row p-value and FDR threshold were set as < 0.05.

Minitab 17 statistical software was used for the qRT-PCR data analysis. Statistical differences were estimated by one-way ANOVA, and the significance of differences among groups was determined by Tukey's t-test. The level of significance was set at a p-value ≤ 0.05. The data are presented as the means ± SD (N = 4).

## Supplementary information

Supplementary information
