# Supplementary material for: Transcriptional and in silico analyses of MIF cytokine and TLR signalling interplay in the LPS inflammatory response of Ciona robusta
Source: Sci Rep. 2020 Jul 9;10:11339. doi: 10.1038/s41598-020-68339-x (PMC7347617; doi:10.1038/s41598-020-68339-x)
Supplement: Supplementary file 1 — Supplementary information [file 41598_2020_68339_MOESM1_ESM.docx]

**Transcriptional and *in silico* analyses of MIF cytokine and TLR signalling interplay in LPS inflammatory response in *Ciona robusta*.**

Vincenzo Arizza^1^, Angela Bonura^2^, Laura La Paglia^3^, Alfonso Urso^3^, Annalisa Pinsino^2^ and Aiti Vizzini^1,*^

^1^Dipartimento di Scienze e Tecnologie Biologiche, Chimiche e Farmaceutiche – Università di Palermo, Via Archirafi 18, Palermo, Italy.

^2^Istituto per la Ricerca e l’Innovazione Biomedica – Consiglio Nazionale delle Ricerche, Via Ugo La Malfa 153, Palermo, Italy.

^3^Istituto di calcolo e Reti ad Alte Prestazioni – Consiglio Nazionale delle Ricerche, Via Ugo La Malfa 153, Palermo, Italy.

 **Figure 1S.** *MyD88* nucleotide and amino acid sequences. The start codon is shown in bold, and the stop codon is indicated by asterisk. The DEATH domain (from 105 to 196 aa) is in orange, and a TIR domain (from 265 to 399 aa) is in green.

**Figure 2S.** Multiple amino acid sequence alignment of MyD88 family members from vertebrates and invertebrates. The DEATH domain is in orange and a TIR domain is in green.

| Gene | Primer sequence (5’-3’) | Application |
| --- | --- | --- |
| *MyD88* | 5’- TGTCTGCTGTTCCTTGGTCA-3’ | RACE5’ |
|  | 5’- TCCTTGGTCAATTGGAGGCT-3’ | NESTED5’ |
|  | 5’- AGCCTCCAATTGACCAAGGA-3’ | RACE3’ |
|  | 5’- TCCTTGGTCAATTGGAGGCT -3’ | NESTED3’ |

**Table 1 S** Primers used for cloning
